# 37889 Taking a pragmatic approach to evaluate Miami Clinical and Translational Science Institute’s Programs using two models.

**DOI:** 10.1017/cts.2021.576

**Published:** 2021-03-30

**Authors:** Rosalina Das, Jessica Diaz, Sheela Dominguez, Barry Issenberg

## Abstract

ABSTRACT IMPACT: Practical evaluation approaches using case studies and success stories present a chain of evidence to demonstrate to stakeholders that resources are being used as required and producing desired results and effectively document the impact of clinical and translational research. OBJECTIVES/GOALS: This project describes the overall evaluation plan of the Miami CTSI by combining the Translational Sciences Benefits Model (TSBM) and the Kirkpatrick Model to evaluate scientific outcomes and impact of CTSI-supported research, and education and training programs developed by the CTSI. METHODS/STUDY POPULATION: Using case studies, the TSBM framework will be applied to CTSI-supported projects to evaluate scientific outcomes and impact on domains that include: clinical and medical; community and public health; economic; legislative and policy. We will apply the framework to projects that have received funding through CTSI’s Pilot and Translational Studies and Mentored Translational Scholars KL2 Programs, and that have at least one publication. Application of the Kirkpatrick model will be demonstrated by using the four levels of evaluation - reaction, learning, behavior, and results - to assess training outcomes and impact of the KL2 and the I-Corps Programs. RESULTS/ANTICIPATED RESULTS: About 20 pilot projects and 8 KL2 research projects will be assessed using the TSBM framework. We anticipate that all projects will show potential or demonstrated benefits in at least two of the four domains of the model. KL2 Program evaluation was conducted by collecting data on all the four levels of the Kirkpatrick model. Reaction and learning were assessed through feedback from KL2 scholars. Behavior was assessed using semi-annual updates on research and training progress of the scholars and the program. Results were measured using indicators such as program graduates that continue to engage in clinical and translational research and their transition to research independence. DISCUSSION/SIGNIFICANCE OF FINDINGS: Our evaluation approach using the two models is well aligned with overall CTSI aims and its three focus areas - infrastructure, education and culturalization/community engagement and will allow us to conduct a comprehensive yet practical evaluation of Miami CTSI programs.

